# Controlled Lattice Thermal Conductivity of Transparent Conductive Oxide Thin Film via Localized Vibration of Doping Atoms

**DOI:** 10.3390/nano11092363

**Published:** 2021-09-11

**Authors:** Young Joong Choi, Ho Yun Lee, Seohan Kim, Pung Keun Song

**Affiliations:** 1 Department of Materials Science and Engineering, Pusan National University, Busan 46241, Korea; yjchoi0782@pusan.ac.kr (Y.J.C.); dbs0770@pusan.ac.kr (H.Y.L.); 2 Department of Materials Science and Engineering, Ångström Laboratory, Uppsala University, 75321 Uppsala, Sweden; 3 Materials Technology and Research, Pusan National University, Busan 46241, Korea

**Keywords:** thermoelectric thin film, transparent conductive oxide, localized vibration, thermal conductivity, magnetron sputtering

## Abstract

Amorphization using impurity doping is a promising approach to improve the thermoelectric properties of tin-doped indium oxide (ITO) thin films. However, an abnormal phenomenon has been observed where an excessive concentration of doped atoms increases the lattice thermal conductivity (*κ*_l_). To elucidate this paradox, we propose two hypotheses: (1) metal hydroxide formation due to the low bond enthalpy energy of O and metal atoms and (2) localized vibration due to excessive impurity doping. To verify these hypotheses, we doped ZnO and CeO_2,_ which have low and high bond enthalpies with oxygen, respectively, into the ITO thin film. Regardless of the bond enthalpy energy, the *κ*_l_ values of the two thin films increased due to excessive doping. Fourier transform infrared spectroscopy was conducted to determine the metal hydroxide formation. There was no significant difference in wave absorbance originating from the OH stretching vibration. Therefore, the increase in *κ*_l_ due to the excessive doping was due to the formation of localized regions in the thin film. These results could be valuable for various applications using other transparent conductive oxides and guide the control of the properties of thin films.

## 1. Introduction

As regulations on carbon emissions are strengthened to prevent global warming, numerous studies of renewable energy are being conducted in various fields to achieve higher energy conversion efficiencies [1,2,3,4,5]. Thermoelectricity is a promising renewable energy source that can replace fossil fuels because it can produce energy through waste heat, unlike other renewable energy sources. To employ thermoelectric (TE) materials in various devices and fields, extensive studies have been carried out to improve the energy conversion efficiency of thin-film thermoelectric materials [6,7,8,9,10,11]. Particularly, as a transparent TE material, tin-doped indium oxide (In_2_O_3_:SnO_2_, ITO) thin film is promising owing to its high electrical conductivity, chemical stability, low toxicity, and low price compared to TE alloys [12,13,14,15]. Accordingly, numerous studies have been carried out to improve the physical properties of ITO, including chemical treatment, impurity doping, and heat treatment, to improve the efficiency compared to opaque TE materials [16,17,18,19,20]. Among them, heat treatment to improve the thin film crystallinity is not promising for ITO-based TE materials because the high crystallinity increases the carrier density (*n*) of the thin film, which results in a decrease in the Seebeck coefficient (*S*) and degrades the thermoelectric figure of merit (ZT) owing to the increased thermal conductivity of electrons. On the other hand, a suitable level of impurity doping can induce a high electrical conductivity without increasing *n* because of the high carrier mobility (*μ*) [17,21,22]. The electrical conduction of ITO is achieved with a conduction band minimum by overlapping of the In 5*s* orbital, which is insensitive to metal-oxide bond angle variation in the amorphous structure [23]. Hence, unlike other materials with directional covalent bonds, such as a-Si, the *μ* of ITO can be maintained in an amorphous thin film by the nondirectional ionic bonding originating from the metal *s* orbital. Regarding metal oxide thin films, which enable a high *μ* in an amorphous structure, impurity-doped amorphous ITO thin films are attractive materials for TE thin films because they can have a comparably low *n*, which decreases the electron thermal conductivity (*κ*_e_) while maintaining a high electrical conductivity (*σ*) owing to the high *μ*.

In our previous study, the microstructure of the ITO thin film was manipulated to control *n* and thermal conductivity without reducing the electrical conductivity of the thin film by doping with ZnO [24]. The total thermal conductivity (*κ*_tot_) decreased with the increase in the ZnO concentration. The highest ZT (0.0627) was achieved with an optimized level of ZnO doping. However, an abnormal phenomenon of lattice thermal conductivity (*κ*_l_) increase was observed when the ZnO concentration exceeded the optimized value, despite the assumption of further progress to a randomly disordered structure proportional to the ZnO concentration. Several studies have been carried out on the increase in *κ*_l_ in relation to the dopant concentration [25,26]. To demonstrate this paradoxical phenomenon, two hypotheses are proposed to explain the *κ*_l_ increase: (1) metal hydroxide formation due to the low bond enthalpy energy of Zn and O and (2) Zn localization due to excessive impurity doping. Further experimental studies are needed to determine the correlation between *κ*_l_ and dopant concentration. To clarify the factor responsible for the increase in *κ*_l_ with a high dopant concentration, an atom that has a high bond enthalpy with O can be employed as a symmetrical dopant.

In this study, we compared the *κ*_l_ values of ZnO- and CeO_2_-doped ITO, which have low and high bond enthalpies with O, respectively (Zn–O: 159 ± 4 kJ/mol, Ce–O: 795 ± 8 kJ/mol). The ITO thin film was amorphized by doping with ZnO or CeO_2_ and exhibited a *σ* and low *κ*_l_ up to the critical level of doped ZnO or CeO_2_. In addition, we investigated the infrared absorbance using Fourier transform infrared (FTIR) spectroscopy to verify whether this phenomenon is due to the formation of metal hydroxide bonds because of the low bond enthalpy energy between the doped atom and oxygen or formation of localized regions in the amorphous material. There was no remarkable difference in the infrared absorbance of the doped thin film, regardless of the different bond enthalpy energies with oxygen. Thus, the increased lattice thermal conductivity due to the excessive doping of ZnO and CeO_2_ originates from localized vibration. We expect that these results can be used for other transparent conductive oxide (TCO)-based thin-film systems and significantly contribute to the development of the TCO thin-film field.

## 2. Materials and Methods

### 2.1. Thin-Film Fabrication

ITO thin films were deposited on a nonalkali glass using a single sintered ITO target (SnO_2_: 10 wt %). ITO:Zn and ITO:Ce thin films were deposited by employing ZnO and CeO_2_ targets and cosputtering with an ITO target (SnO_2_: 10 wt %) via a magnetron cosputtering system on the nonalkali glass. The base pressure of the deposition process was 1.5 × 10^−5^ Torr. The total gas pressure was maintained at 1.0 Pa using an Ar gas flow of 20 sccm. All the thin-film samples were deposited to a thickness of 150 nm.

### 2.2. Thin-Film Characterization

The microstructures of the thin films were analyzed using X-ray diffraction (XRD; D8 Advance, Bruker, Billerica, MA, USA). A Hall effect measurement system (HMS2000, Ecopia, Anyang, Korea) was employed to measure the electrical properties of the thin films. The transmittance was estimated using an ultraviolet (UV)–visible analysis (UV-1800, Shimadzu, Kyoto, Japan). The *κ*_tot_ value of the thin film was measured using the time-domain thermoreflectance (TDTR) method, which uses a 765 nm Ti:sapphire laser. An Al thin film of 85 nm thickness was deposited on the ITO:Zn and an ITO:Ce thin film of 150 nm thickness as the thermal transducer layer of a femtosecond pulsed light beam. The volumetric heat capacity of ITO:Zn, ITO:Ce, and the Al thin film was assumed to be the same as ITO and Al bulk materials [27,28]. The *κ*_e_ value was calculated using the equation *κ*_e_ = *L*_0_*T*/*ρ* via the carrier density obtained by Hall measurements, where *L*_0_, *T*, and *ρ* are the Lorentz coefficient, absolute temperature, and resistivity, respectively. After the measurement and calculation, *κ*_l_ was obtained by subtracting *κ*_e_ from *κ*_tot_. Finally, the OH stretching vibration of the doped ITO thin film was measured using FTIR spectroscopy (Vertex 80v, Bruker, Billerica, MA, USA).

## 3. Results and Discussion

**Figure 1:**
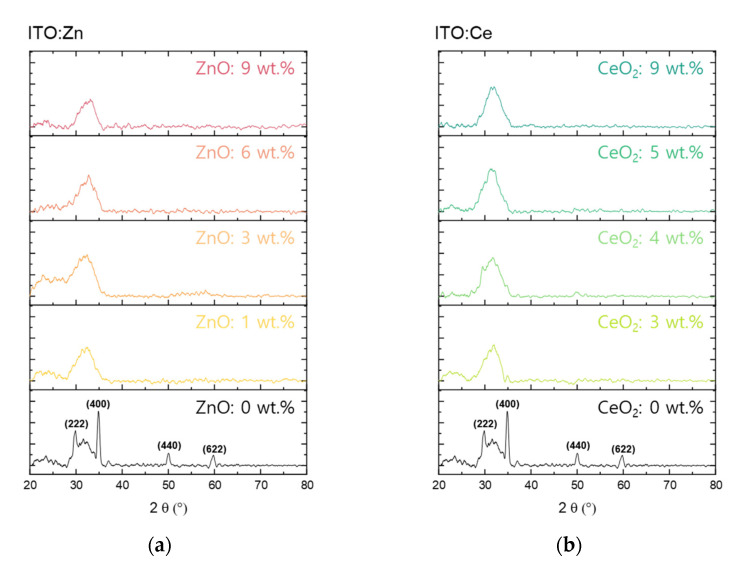
XRD patterns of (**a**) ZnO-doped ITO and (**b**) CeO_2_-doped ITO. The pure ITO exhibits a polycrystalline structure. The crystal structure of ITO doped with ZnO and CeO_2_ becomes amorphous even with a small level of doping. There is no change in the diffraction pattern when the doping amount is further increased.

To analyze the influence of the dopant material in the ITO thin film on the crystallinity of the thin film, ZnO (ITO:Zn) and CeO_2_ (ITO:Ce) were deposited on a nonalkali glass substrate. As shown in Figure 1, the undoped ITO thin film exhibits the preferred orientations (222) and (400), which indicates that the pure ITO has a polycrystalline structure.

In the case of the doped ITO (ITO:Zn or ITO:Ce), both thin films exhibited amorphous structures regardless of the doping amount. With the increase in the doping amount of ZnO and CeO_2_, the main peaks of (222) and (400) of ITO disappeared and became broad, which indicates that the thin film transformed the microstructure from polycrystalline to amorphous. Despite the bond enthalpy energy difference of the doped atom with oxygen, the ITO thin film was amorphized even with a small amount of doped ZnO and CeO_2_.

**Figure 2:**
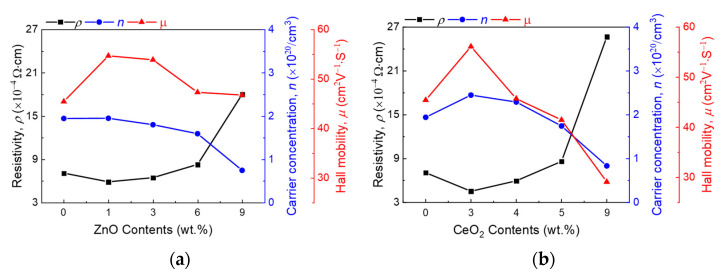
Comparison of electrical properties of (**a**) ZnO-doped ITO and (**b**) CeO_2_-doped ITO depending on the concentration of doping atoms. With a smaller amount of doping, the resistivities of both thin films decrease. On the other hand, excessive doping degrades the electrical properties of the thin film.

The electrical properties of the ITO:Zn and ITO:Ce thin films are shown in Figure 2. In the case of ITO:Zn, the lowest resistivity (*ρ*) was observed for 1 wt % of ZnO owing to the formation of an amorphous-like ternary compound. At concentrations higher than 1 wt %, the *n* of the ITO:Zn thin film decreased as a function of the ZnO concentration [16,29,30]. In the case of CeO_2_, the *ρ* of the ITO:Ce thin film decreased up to 3 wt %; above this concentration, *ρ* increased. Despite the difference in bond enthalpy with oxygen, both ITO samples exhibited typical electrical property trends in relation to the dopant concentration.

**Figure 3:**
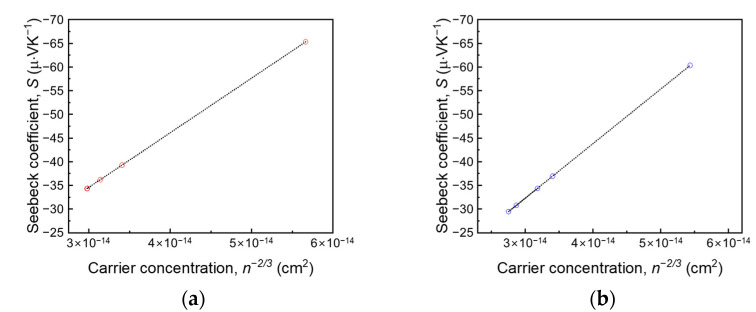
Seebeck coefficients (*S*) and *n*^−(2/3)^ of (**a**) ZnO-doped ITO and (**b**) CeO_2_-doped ITO at various dopant concentrations. The Mott equation for metals and degenerated semiconductors was employed to calculate *S* using the measured carrier density.

The *S* value was calculated using the Mott equation with the measured *n* to analyze the effect of Zn and Ce atoms with different bond enthalpy energies on the thermoelectric performance of the thin film. For metal and degenerated semiconductors, the Mott equation is

(1) $$S=\frac{8\pi^{2}k_{B}{}^{2}}{3eh^{2}}{\left(\frac{\pi }{3n}\right)}^{\frac{2}{3}}m^{*}T,$$

where *k*_B_, *h*, m*, *e*, *n*, and *T* are the Boltzmann constant, Planck’s constant, effective mass (0.561*m_e_* = 5.110 × 10^−31^ kg), electron charge, majority charge carrier density, and absolute temperature, respectively [31]. As shown in Equation (1), the *S* value is proportional to *n*^−2/3^. Figure 3 shows *S* values plotted in relation to *n*^−2/3^, and it shows a linear proportional relationship with respect to *n*^−2/3^. The ITO:Zn thin film has a lower *n* than that of ITO:Ce, which leads to a relatively higher *S* at a ZnO content of 1 wt %. The highest *S* was obtained at 9 wt % owing to the considerable decrease in *n*. With the decrease in *n* owing to the increased doping amount of CeO_2_, ITO:Ce exhibited a relatively low *S* at 9 wt %.

**Figure 4:**
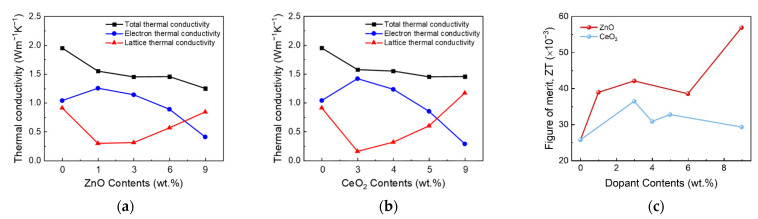
Dependence of the thermal conductivities of the (**a**) ZnO-doped ITO and (**b**) CeO_2_-doped ITO on the doping concentration. (**c**) Thermoelectric figures of merit (ZT) of ZnO- and CeO_2_-doped ITO. The lattice thermal conductivity increased above a certain doping concentration.

Figure 4 shows the *κ*_tot_, *κ*_e_, and *κ*_l_ values of (a) ITO:Zn, (b) ITO:Ce, and (c) ZT of the ITO:Zn and ITO:Ce thin films. The ZT values were calculated using

ZT = *σS*^2^*T*/*κ*_tot_, (2)

where *σ*, *S*, *T*, and *κ*_tot_ are the electrical conductivity, Seebeck coefficient, absolute temperature, and total thermal conductivity, respectively. The highest ZT (0.0558) was obtained at the highest ZnO dopant concentration owing to the low thermal conductivity. These results indicate relatively high thermoelectric performance in the recently reported TCO-based n-type thermoelectric material [7,9,32,33]. The *κ*_l_ value was obtained by *κ*_tot_ and *κ*_e_, calculated using *κ*_e_ = *L*_0_*T*/*ρ*, where *L*_0_, *T*, and *ρ* are the Lorentz coefficient, absolute temperature, and resistivity, respectively. The *κ*_l_ value can then be obtained using *κ*_tot_ = *κ*_l_ + *κ*_e_. Regardless of the dopant atom, *κ*_tot_ decreased with the increase in the doping concentration. These trends are affected by the weakened electrical properties and amorphization of the microstructure.

However, an abnormal phenomenon of *κ*_l_ increase above certain dopant concentrations was observed for both ZnO- and CeO_2_-doped ITO. As mentioned previously, the impurity doping in thin films causes disordered crystallinity, which deteriorates *κ*_l_. Therefore, *κ*_l_ should also be decreased; however, above a certain concentration, *κ*_l_ is slightly increased. We propose two hypotheses to explain the *κ*_l_ increase: (1) metal hydroxide formation and (2) localization of excessive dopant atoms.

**Figure 5:**
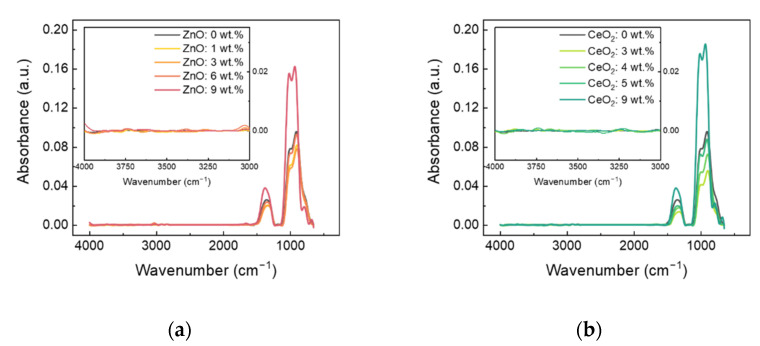
FTIR spectroscopy of (**a**) ZnO-doped ITO and (**b**) CeO_2_-doped ITO. The inset shows a detail of the OH stretching vibration area. Although Zn and Ce have different bond enthalpies with oxygen, there is no change in the OH stretching vibration region according to the doping amount.

Figure 5 shows the FTIR spectroscopy results for (a) ITO:Zn and (b) ITO:Ce to verify the presence of metal hydroxide in the doped ITO in relation to the dopant material. The FTIR spectroscopy analysis showed wavenumbers of 650 to 4000 cm^−1^. The peaks at 1023.49–1026.37 cm^−1^ are related to the phonon mode of the In_2_O_3_ lattice. The OH stretching vibration mode is related to the wavenumber of approximately 3240 cm^−1^ [34,35,36,37,38,39]. The wavenumber region of 650 to 1500 cm^−1^ corresponds to the typical signal of the ITO thin film, without significant difference, especially near the 1500 cm^−1^ peak, which originates from O–H bending vibration of absorption water [40,41,42]. In addition, the OH stretching vibration region (wavenumbers around 3240 cm^−1^) did not show any signals for either ITO:Zn or ITO:Ce thin films despite a high doping concentration of ZnO and CeO_2_ (9 wt %). This provides clues that the *κ*_l_ increase above the specific dopant concentration is not related to metal hydroxide formation but originates from localized vibration due to the excessive dopant amount. Therefore, the localized region generated by excessive doping of ZnO and CeO_2_ becomes a new pathway for heat transfer in ITO and suppresses phonon scattering by cations, leading to the increase in *κ*_l_ [25,43].

## 4. Conclusions

In this study, we prepared ITO:Zn and ITO:Ce thin films with various dopant concentrations to verify the reason for the increase in *κ*_l_ above a certain dopant concentration. The proposed hypotheses were (1) metal hydroxide formation and (2) localized impurity vibration. We employed Zn and Ce, with low and high bond enthalpies with oxygen atoms, respectively. The pure ITO thin film exhibited a polycrystalline structure, while, regardless of the dopant atom, the doped ITO exhibited an amorphous structure. Although there was a difference between the bond enthalpy energies of Zn and Ce with O, the electrical properties of the thin films deteriorated owing to the decrease in *n* originating from the doping in both ITO:Zn and ITO:Ce. Both ITO:Zn and ITO:Ce exhibited the lowest thermal conductivities at 9 wt % because of the reduced *κ*_e_ due to the significant *n* reduction. The *κ*_l_ value was increased by excessive doping regardless of the bond enthalpy energy, which indicates that the bond enthalpy does not affect the increase in *κ*_l_. The reason for the increase in *κ*_l_ was determined by the FTIR spectroscopy results; there was no notable difference in the wavenumber of the OH stretching vibration. The wavenumber region of the OH stretching vibration area did not exhibit any signal, which indicated that the increase in *κ*_l_ did not originate from metal hydroxide formation but from localized vibration with the excessive doping of atoms. We think that these results will guide the future control of the properties of thin films and contribute to next-generation thin-film applications.

## Data Availability

Data is contained within the article.
